# Recurrent Bacteremia in the Setting of a Coronary Artery Fistula

**DOI:** 10.7759/cureus.9289

**Published:** 2020-07-20

**Authors:** Khushali Shah, Yash Jobanputra, Purva Sharma

**Affiliations:** 1 Internal Medicine, University of Miami Miller School of Medicine, Miami, USA; 2 Internal Medicine, University of Miami Miller School of Medicine, Atlantis, USA

**Keywords:** congenital anomalies, cardiac imaging, bacteremia

## Abstract

We present an interesting case of a 31-year-old female with recurrent *Staphylococcus lugdunensis* bacteremia in the setting of a coronary artery fistula (CAF). Over the course of several months, the patient was admitted to the hospital on three separate occasions with an unclear source of bacteremia. She suffered from numerous complications, including cavitary pneumonia, osteomyelitis, synovitis and septic emboli. On each admission, the patient received intravenous (IV) antibiotic therapy. CT scan of the chest with contrast on the third admission revealed a prominent tortuous vessel coursing from the ascending aorta and main pulmonary artery to the left atrium. Coronary CT angiogram confirmed the presence of a fistula connecting the left circumflex artery to the coronary sinus. Common complications of CAF include infective endocarditis and myocardial ischemia; however, we report a novel case of recurrent bacteremia in the context of an anomalous coronary artery. Two months after diagnosis, surgical closure of the CAF was performed. This case illustrates the importance of utilizing different cardiac imaging modalities in order to diagnose congenital cardiac anomalies in a timely fashion and intervene appropriately.

## Introduction

Coronary arteriovenous fistulas (CAF) are congenital or acquired abnormalities characterized by an abnormal connection between the coronary circulation and cardiac chambers and/or other great vessels [1]. Most patients with CAF are asymptomatic and are typically found incidentally by performing coronary angiography, CT angiography or transthoracic echocardiography (TTE). Complications of CAF include myocardial ischemia, infective endocarditis and heart failure secondary to shunting [2]. We present a rare case of recurrent bacteremia in a symptomatic young adult woman found to have a CAF.

## Case presentation

A 31-year-old Caucasian female with no significant past medical history presented to the emergency department with high-grade fevers, chills and right upper quadrant abdominal pain two days after an uneventful laparoscopic cholecystectomy for acute cholecystitis. On presentation, she was febrile (100.8°F) without evidence of tachycardia or tachypnea. The remainder of her physical examination was unremarkable. Laboratory studies revealed a white blood cell count on admission of 3.5 K/µL trending downward to 2.3 K/µL over the course of her five-day hospital stay. CT of the abdomen and pelvis showed trace fluid in the pelvis without evidence of solid organ injury. She was started on empiric intravenous (IV) piperacillin-tazobactam. Two sets of blood cultures grew pansensitive* Staphylococcus lugdunensis*, and she was subsequently switched to IV vancomycin for better coverage. The source of the bacteremia was unclear; however, within two to five days repeat cultures were negative; sepsis and leukopenia resolved, and the patient was discharged on oral levofloxacin with instructions to complete the course of her antibiotic therapy.

The patient developed fevers and chills again two months later in addition to left clavicular swelling and tenderness. Similar to her previous admission, she again grew two/two sets of *S. lugdunensis* on blood cultures. On physical examination, the patient had decreased breath sounds, and CT scan of her chest revealed two new left-sided cavitary lung lesions (largest measuring 17 mm), attributed to an infectious etiology. Pulmonary tuberculosis was ruled out with negative QuantiFERON® Gold assay and two negative sputum cultures for acid-fast bacilli. The patient was started on IV cefazolin. She was diagnosed with left sternoclavicular joint synovitis and early osteomyelitis by chest MRI; however, the source of the pulmonary cavitary lesion remained unidentified. To rule out endocarditis with septic emboli, TTE and transesophageal echocardiography (TEE) were conducted. Both studies did not reveal any valvular vegetations and/or structural valve abnormalities. The patient also denied any use of IV drugs. She was diagnosed with cavitary pneumonia from bacteremia, which had improved since admission with antibiotic therapy. She was discharged with a peripherally inserted central catheter (PICC) line and outpatient IV cefazolin for a total of four weeks.

**Figure 1:**
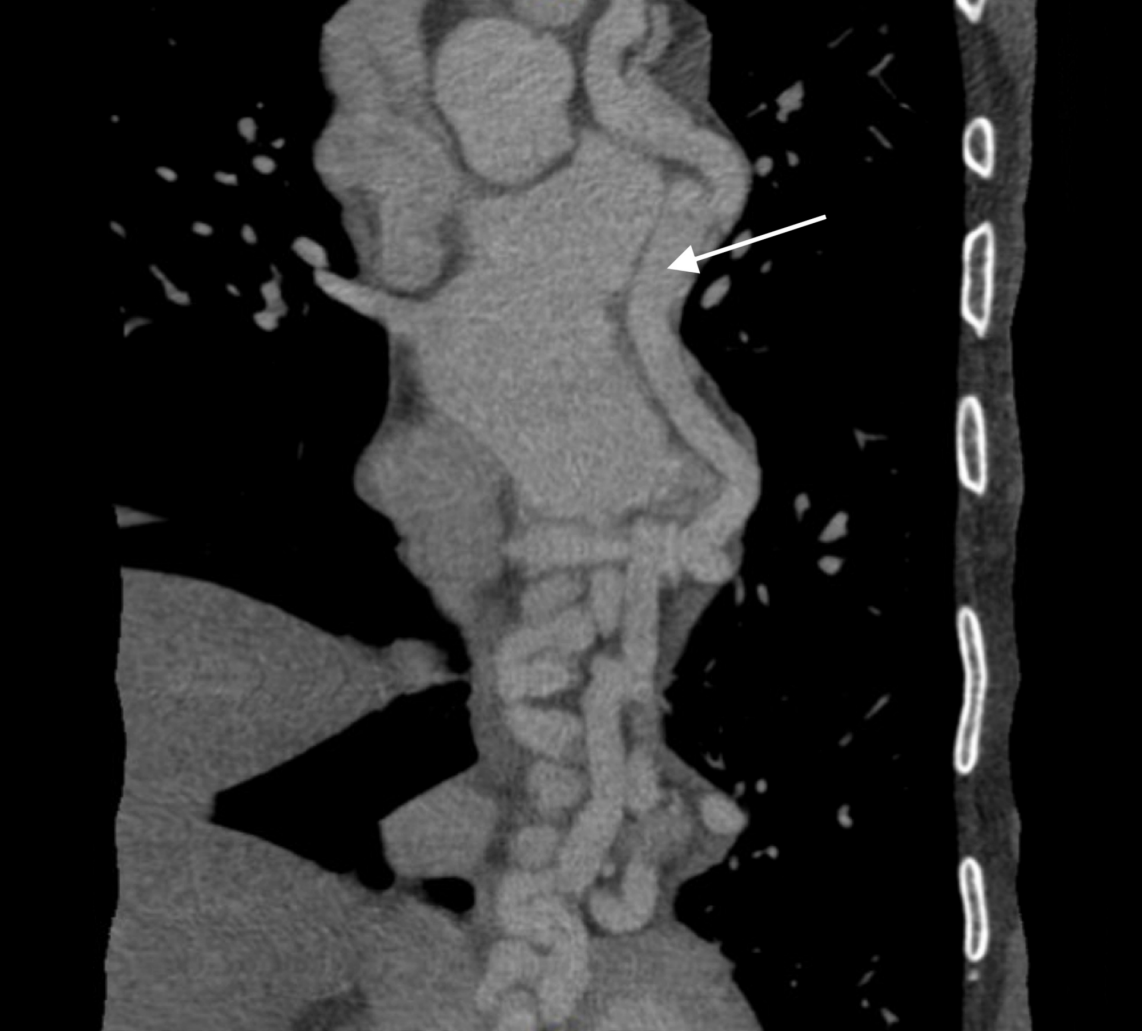
Coronary CT angiogram showing fistula extending from left circumflex artery (LCX) to coronary sinus (white arrow).

A month after the patient completed the duration of her antibiotic therapy, she began to experience subjective fevers, fatigue and low back pain. Blood cultures grew *S. lugdunensis* for the third time. TTE was repeated, which showed no evidence of vegetation. CT scan of the chest with contrast showed a new right middle lobe opacity with some cavitation consistent with an infectious etiology, and the patient was again started on an IV cephalosporin antibiotic. The scan also showed an incidental finding of a prominent tortuous vessel coursing from the vicinity of the ascending aorta and main pulmonary artery to the left atrium. This finding was suggestive of either a CAF or anomalous left coronary artery arising from the pulmonary artery (ALCAPA). Coronary CT angiogram was done, which showed, more specifically, the presence of a fistula connecting the left circumflex artery (LCX) to the coronary sinus (Figure 1). It was hypothesized that the recurrent bacteremia and septic emboli to the lung were likely due to the presence of this fistula. The patient was referred to a congenital cardiac disease specialist for further work-up and repair of the fistula. Surgical closure of the CAF was performed two months later, and the patient has not had any further complications since the surgery.

## Discussion

The diagnosis of a coronary artery anomaly requires a high index of suspicion when conducting a history and physical examination. Non-specific presentations are common, which makes diagnosis and management particularly challenging. Anomalous coronary arteries can, however, present with life-threatening conditions, such as myocardial infarction or septic bacteremia. Cardiac catheterization remains the gold standard for diagnosis of a coronary artery anomaly. Recognition of important clues and specific angiographic views, however, are required to fully delineate such anomalies. Some of the known complications of CAFs include infective endocarditis with valvular vegetations resistant to antibiotics, atherosclerotic deposition, myocardial ischemia, congestive heart failure, aneurysmal dilation and a left-to-right shunt with progressive dilation of both ventricles [3-5].

A majority (35%-40%) of CAFs originate from the left anterior descending artery, whereas 5% to 20% originate from the LCX [6]. Drainage site in the coronary sinus has an even lower incidence rate of 7%, as reported in our patient’s case [6]. There was absence of vegetations on TTE/TEE in our patient, which eliminated endocarditis from the differential. Our patient’s clinical course, however, was complicated by septic pulmonary emboli and osteomyelitis secondary to persistent *S. lugdunensis* non-infective endocarditis bacteremia resistant to antibiotics. *S. lugdunensis *is a coagulase-negative *Staphylococcus *with presentation ranging from harmless skin flora to severe life-threatening infection [7,8]. Interestingly, *S. lugdunensis *when classified as life-threatening often presents with infective endocarditis but in our patient, the virulent bacteria took a different course: bacteremia marked by re-appearance on three separate occasions. Surgical correction of the fistula once the patient was clinically stable was thought to be the only definitive management option.

The evolution of CAF has a unique history as earlier studies indicated anomalies stemming from the right coronary system, whereas more recent studies reveal the left coronary system as the site of origin [4]. Our patient had a presentation similar to the latter but with the added nuance of recurrent bacteremia. As medicine continues to advance, developments in the field of cardiac imaging aid in the diagnosis of complex medical conditions such as CAF. A case report by Punzo et al suggested the need to make use of advanced non-invasive imaging modalities such as cardiac magnetic resonance and cardiac computerized tomography to provide a better understanding of prognosis [6]. In addition, this sophisticated technology more accurately depicts cardiac function, morphology and tissue characterization to ultimately direct management and treatment.

The incidence of recurrent bacteremia with concomitant complications in the presence of CAF is unknown. To the best of our knowledge, this is the first case report outlining septic pulmonary emboli in the absence of infective endocarditis but rather secondary to recurrent bacteremia from a CAF. It is always important to consider congenital cardiac anomalies such as CAF in patients with recurrent bacteremia and no revealing source after preliminary work-up. Early diagnosis prevents secondary complications of bacteremia, such as deep tissue infections and pulmonary cavitation. Although the literature cites prophylactic antibiotic treatment of infective endocarditis in patients diagnosed with CAF, there are currently no recommended guidelines for the treatment of recurrent bacteremia in the setting of CAF. Prior to discovery of the congenital cardiac anomaly on final hospitalization, we followed the guidelines for treating isolated recurrent bacteremia. Once cardiac imaging confirmed the congenital anomaly, however, surgical management was suggested. A recent article by Ali et al. outlines an algorithm for the management of coronary fistula either by transcatheter intervention or a more invasive surgical route based on symptoms, size, location and characterization of the fistula [9].

## Conclusions

This is a case of recurrent bacteremia with an initially unidentified source presenting with several complications up until an anomalous cardiac fistula was incidentally detected. This case highlights the importance of performing advanced cardiac imaging on patients with an unknown etiology for recurrent bacteremia after exhausting conventional testing methods. Establishing the correct diagnosis early in the course of disease can remedy unnecessary medical complications. The advances in technologies permit various modes of imaging that help detail cardiac tissue reserve, function and morphology utilized to guide decision-making and treatment. The expanded use of non-invasive transcatheter intervention or surgical repair of a CAF warrants further investigation.
